# Characterization of Selenium Accumulation, Localization and Speciation in Buckwheat–Implications for Biofortification

**DOI:** 10.3389/fpls.2018.01583

**Published:** 2018-10-31

**Authors:** Ying Jiang, Ali F. El Mehdawi, Tripti, Leonardo W. Lima, Gavin Stonehouse, Sirine C. Fakra, Yuegao Hu, Hua Qi, Elizabeth A. H. Pilon-Smits

**Affiliations:** ^1^College of Agronomy, Shenyang Agricultural University, Shenyang, China; ^2^Department of Biology, Colorado State University, Fort Collins, CO, United States; ^3^College of Agronomy and Biotechnology, China Agricultural University, Beijing, China; ^4^Department of Experimental Biology and Biotechnology, Institute of Natural Sciences and Mathematics, Ural Federal University, Ekaterinburg, Russia; ^5^Advanced Light Source, Lawrence Berkeley National Laboratory, Berkeley, CA, United States

**Keywords:** common buckwheat, tartary buckwheat, selenite, selenate, biofortification, X-ray microprobe

## Abstract

Buckwheat is an important crop species in areas of selenium (Se) deficiency. To obtain better insight into their Se metabolic properties, common buckwheat (*Fagopyrum esculentum*) and tartary buckwheat (*F. tataricum*) were supplied with different concentrations of Se, supplied as selenate, selenite, or *Astragalus bisulcatus* plant extract (methyl-selenocysteine). Se was supplied at different developmental stages, with different durations, and in the presence or absence of potentially competing ions, sulfate, and phosphate. The plants were analyzed for growth, Se uptake, translocation, accumulation, as well as for Se localization and chemical speciation in the seed. Plants of both buckwheat species were supplied with 20 μM of either of the three forms of Se twice over their growth period. Both species accumulated 15–40 mg Se kg^−1^ DW in seeds, leaves and stems, from all three selenocompounds. X-ray microprobe analysis showed that the Se in seeds was localized in the embryo, in organic C-Se-C form(s) resembling selenomethionine, methyl-selenocysteine, and γ-glutamyl-methylselenocysteine standards. In short-term (2 and 24 h) Se uptake studies, both buckwheat species showed higher Se uptake rate and shoot Se accumulation when supplied with plant extract (methyl-selenocysteine), compared to selenite or selenate. In long-term (7 days) uptake studies, both species were resistant to selenite up to 50 μM. Tartary buckwheat was also resistant to selenate up to 75 μM Se, but >30 μM selenate inhibited common buckwheat growth. Selenium accumulation was similar in both species. When selenite was supplied, Se levels were 10–20-fold higher in root (up to 900 mg Se kg^−1^ DW) than shoot, but 4-fold higher in shoot (up to 1,200 mg Se kg^−1^ DW) than root for selenate-supplied plants. Additionally, sulfate and phosphate supply affected Se uptake, and conversely selenate enhanced S and P accumulation in both species. These findings have relevance for crop Se biofortification applications.

## Introduction

For humans and other mammals, selenium (Se) is an essential micronutrient involved in a number of essential functions, including fertility, thyroid, and immune function; hence, Se deficiency enhances the risk of developing cancers and infections (Rayman, 2012). The recommended daily intake for adult humans is 50–70 μg Se per day (Bendich, 2001). Based on recent studies, 0.5 to 1 billion people may be suffering from diseases that might be caused by Se deficiency, due to low soil Se levels (Combs, 2001; Jones et al., 2017). This Se deficiency is predicted to worsen due to reducing soil Se levels under global climate change, especially in low-Se areas in China and Europe (Jones et al., 2017). For this reason, Se deficiency issues have been attracting an increasing focus worldwide. An additional complication is that Se can become toxic at high levels, and there is only a narrow window (less than an order of magnitude) between Se deficiency and toxicity (Stadtman, 1974).

Selenium's essentiality for plants has not been established, but Se is considered a beneficial element that enhances plant growth (Sors et al., 2005; Schiavon and Pilon-Smits, 2017). As one of the dominant sources of dietary Se intake worldwide, production of Se-fortified crops has been recognized as a strategy to cope with the issue of Se deficiency (Jiang et al., 2015; Schiavon et al., 2017). A common strategy is to biofortify plants with Se via fertilization with inorganic or organic forms of Se (Broadley et al., 2006; Schiavon and Pilon-Smits, 2017). To optimize Se accumulation and speciation in different crop species, it is important to characterize uptake and translocation patterns of different forms of Se, particularly in the edible parts.

Selenate and selenite are the two dominant forms of inorganic Se available for plant uptake in natural conditions (White et al., 2007). Organic forms also occur in nature, when organisms reductively assimilate selenate or selenite via the sulfate assimilation pathway (Terry et al., 2000). Different plant species differ in the extent to which they assimilate Se. This is relevant for biofortification, because organic forms of Se are considered a better form of dietary Se (Navarro-Alarcon and Cabrera-Vique, 2008). A potentially interesting source of Se for crop fertilization is green manure from Se hyperaccumulator plant, i.e., plants that naturally hyperaccumulate Se in organic form (mainly methyl-selenocysteine, methyl-SeCys) up to 1% of dry weight (Yasin et al., 2014). Hyperaccumulator plants have been successfully applied as a source of organic Se for crop Se biofortification (Bañuelos et al., 2016).

There are substantial differences between the mechanisms involved in plant uptake and translocation of selenate, selenite, and organic forms Se, as reviewed by Sors et al. (2005). The bioavailability and uptake of environmental Se differs with environmental conditions, Se species and plant species (Zhu et al., 2009). Sulfate competitively inhibits selenate uptake by plant roots, since they make use of the same transporter and metabolic pathway (Schiavon and Pilon-Smits, 2017). Controversies still exist regarding selenite uptake by plants; it has been suggested that this is likely a passive process, but it may also make use of phosphate transporters (Li et al., 2008; Zhang et al., 2014). In addition to inorganic Se forms, organic forms of Se can be taken up by plants (Abrams et al., 1990), and often at much higher rates than the uptake of inorganic species (de Souza et al., 2000; Kikkert and Berkelaar, 2013; Yasin et al., 2014). To date, although Se hyperaccumulation plants have been utilized as a Se source for crop biofortification (Bañuelos et al., 2015, 2016), little information is still available about Se uptake and translocation patterns of Se derived from Se hyperaccumulation plants. Thus, a comparative study of Se uptake in the forms of selenate, selenite, and hyperaccumulator-derived organic Se forms is worth further investigation. Better knowledge of the uptake and translocation of hyperaccumulator-derived Se by crop species will be directly applicable for biofortification (Graham et al., 2007).

Different crop species vary in their Se accumulation, metabolism, partitioning and tolerance, and within each species Se accumulation and speciation can also differ with growth stage and plant organ (Hasanuzzaman et al., 2010). Buckwheat is emerging as a very important alternative crop in areas of Europe and Asia where Se-deficiency commonly occurs. As shown in previous field studies, buckwheat has substantial Se accumulation capacity and may be a suitable crop species for Se biofortification (Jiang et al., 2015; Golob et al., 2016). However, Se uptake and translocation of buckwheat has not been well-characterized under controlled conditions. In the current study, therefore, Se uptake and translocation were characterized in two species of buckwheat supplied with selenate, selenite or methyl-SeCys containing plant extract from Se hyperaccumulator *Astragalus bisulcatus*. Additionally, the effects of sulfate and phosphate on uptake of these Se species were investigated. The Se species were supplied at a range of concentrations, so as to investigate not only which Se supply is optimal for Se biofortification, but also to determine Se tolerance. Furthermore, x-ray microprobe analysis was used to determine the form and localization of Se in buckwheat seeds of both species supplied with selenate, selenite, or methyl-SeCys.

## Methods and materials

### Plant material and growth conditions

Common buckwheat (*Fagopyrum esculentum* Moench) and tartary buckwheat (*Fagopyrum tataricum* Gaertn.) seeds were surface sterilized by rinsing for 20 min in 20% bleach, followed by five 10-min rinses in sterile water and stratified at 4°C for 3 days before sowing. Then the seeds were sown and allowed to germinate in Turface®/sand (2:1) mixture. The plants were cultivated inside a growth chamber at 24°C under fluorescent lights at a 16 h:8 h light: dark photoperiod. Plant extract used in the current study was from leaves of Se hyperaccumulator *Astragalus bisulcatus* which were collected from the seleniferous Pine Ridge Natural Area in Colorado, described previously (El Mehdawi et al., 2012). After grinding of the fresh leaves in liquid nitrogen, 4 mL g^−1^ fresh weight of acidic deionized water (pH 2.5) was used to extract the Se from the pulverized plant sample. After 1 h extraction on ice, with occasional mixing, the supernatant was collected through centrifugation at 2,500 × g for 10 min, and frozen at −20°C until use. The main form of Se in this plant extract was confirmed via liquid chromatography-mass spectrometry (not shown) to be methylselenocysteine (methyl-SeCys), with a minor fraction of γ-glutamyl-methyl-SeCys, as reported before (Valdez Barillas et al., 2012). The Se concentration in the extract was determined using Inductively coupled plasma optical emission spectroscopy (ICP-OES) as described below.

### Selenium uptake experiment

In order to investigate the uptake capacity of common buckwheat and tartary buckwheat for different forms of Se (selenate, selenite, and plant-extracted methyl-SeCys), and the interactions of sulfate and phosphate with uptake of these different Se species, 1-week old buckwheat plants were transplanted into 5–l hydroponic containers with 1/4-strength Hoagland nutrient solution (Hoagland and Arnon, 1950) and cultivated for 1 week before the uptake experiment. The uptake experiment was performed in 100-ml containers with one plant per container with three replications for each treatment. After rinsing with deionized water, the plants were incubated for 2 h in 20 μM Se and 2 mM MES buffer (pH 5.6) with/without 0.5 mM sulfate for selenate uptake, with/without 0.5 mM phosphate for selenite uptake, and with/without 0.5 mM sulfate or phosphate for plant extract methyl-SeCys uptake. After the 2 h uptake period, the plants were incubated in ice-cold 2 mM CaCl_2_ and 2 mM MES (pH 5.6) desorption solution for 2 min to remove Se from the root apoplast. Then the plants were blotted dry, shoots and roots were separated, dried, weighed, and analyzed for elemental concentrations as described below.

### Selenium tolerance experiment

Seeds of the two buckwheat species were sown into 50 ml pots with Turface®/sand (2:1) mixture. The pots were placed in 100 ml containers and supplied with 1/4-strength Hoagland nutrient solution. When the seedlings were 1 week old, they were thinned out to 2 plants per pot. After 1 more week of cultivation, the plants were incubated for 7 days in 1/4-strength Hoagland nutrient solution with the presence of a series of concentration of selenate (0, 15, 30, 45, 60, 75 μM Na_2_SeO_4_) or selenite (0, 10, 20, 30, 40, 50 μM Na_2_SeO_3_) to determine the Se accumulation and tolerance. After harvest, the roots were rinsed with deionized water and soaked for 2 min in ice-cold desorption solution (2 mM CaCl_2_ and 2 mM MES pH 5.6). Then the plants were blotted dry, separated into shoot and root, and dried. The plant organs were then weighed and analyzed for elemental composition as described below.

### Fate of selenium in mature plants after two-time se biofortification

The pre-sterilized seeds from both buckwheat species were sown in 1 L pots with Turface®/sand (2:1, w/w) mixture, thinned to 2 plants per pot when the plants were 1-week old, and grown until maturity, supplied with 1/4-strength Hoagland nutrient solution. When the plants were 30 days old, they were divided into four groups of four pots each. One group was kept as control, while the other three were supplied with 20 μM Se from either Na_2_SeO_4(_selenate), Na_2_SeO_3_ (selenite) or plant extract (methyl-SeCys). The Se was supplied twice, first at 30 d and again at 45 d of age. When most of the seeds had matured from both buckwheat species, the plants were harvested and separated into organs (root, stem, leaf, and grain). The roots were rinsed with deionized water and blotted dry. Some seeds were stored at −80°C for X-ray microprobe analysis as described below. Subsequently, all organs were dried, weighed and used for elemental analysis as described below.

### Comparison of selenite, selenate and plant extract se uptake and assimilation in common buckwheat and tartary buckwheat for 24 h uptake assay

Thirty-day-old buckwheat plants were grown in Turface® in 1 L pots (two plants per pot), supplied with 1/4-strength Hoagland nutrient solution. Twenty μM of Se was supplied as Na_2_SeO_3_, Na_2_SeO_4_ or plant extract (methyl-SeCys). Each treatment was replicated in three pots. After 24 h of Se supplement, the collection of xylem sap was carried out as described by Li et al. (2008). The shoots were cut at 2 cm above the roots. Deionized water was used to rinse the cut surfaces, then the surface was blotted dry, and xylem sap was collected with a pipette over the following 2 h. Roots were rinsed with deionized water and then soaked in an ice-cold 2 mM CaCl_2_ and 2 mM MES (pH 5.6) solution for 10 min desorption. Root and shoot samples were used for the determination of total Se. The xylem sap was stored in −80°C before analysis via LC-MS.

### Elemental analysis

Tissues and organs of buckwheat plants were dried at 50°C in the oven until constant weight and then digested in nitric acid as described by Zarcinas et al. (1987). Inductively coupled plasma optical emission spectroscopy (ICP-OES) was used as described by Fassel (1978) to measure Se, S, and P concentrations in the digests, using appropriate quality controls and standards. The chemical speciation of Se in xylem sap was detected using liquid chromatography-mass spectrometry (LC-MS) as documented by Dumont et al. (2006), using a mixture of organic Se compounds as standards.

### Statistical analysis

The software SAS (v.9.2; SAS Institute, USA) was used for statistical data analysis. The Duncan's multiple rank test was used to compare means of traits at *P* = 0.05. All datasets were tested for normal distribution and equal variance. For calculation of Se uptake rate per g root DW over the 2 h experiment, the total amount of Se accumulated in the plant was divided by root DW, via the equation ([*Se*]_root_×*DW*_root_+[*Se*]_shoot_×*DW*_shoot_)/Root DW. The translocation factor (TF) was calculated as the ratio of the total amount of Se in shoot ([Se]_shoot_×*DW*_shoot_) to the total amount of Se in root ([Se]_root_×*DW*_root_), as described by Kikkert and Berkelaar (2013).

### X-Ray microprobe analysis

Seeds obtained from plants supplied twice during their lifetime with 20 μM Se as selenate, selenite or *A. bisulcatus* plant extract were shipped on dry ice and mounted on a Peltier stage kept at −25°C during analysis. X-ray microprobe analyses were performed at beamline 10.3.2 of the Advanced Light Source, at Lawrence Berkeley National Lab (Berkeley, USA) (Marcus et al., 2004). Se, Ca, K spatial distribution in the samples were determined using μXRF mapping at 13 keV incident beam, using a pixel size of 20 × 20 μm and a beam size of 5 × 5 μm. Maps were subsequently deadtime-corrected and decontaminated. In specific sample regions of interest, chemical speciation of Se was determined using Se K-edge μXANES spectroscopy, following procedures previously described in El Mehdawi et al. (2012). Se spectra and XRF maps were recorded in fluorescence mode using a Ge solid state detector. Spectra were calibrated using a red amorphous Se standard, with the main peak set at 12660 eV. All spectra recorded in the range 12500–13070eV, were deadtime-corrected, deglitched, calibrated, pre-edge background substracted, and post-edge normalized using a suite of custom LabVIEW software available at the beamline. Least-squares linear combination fitting (LCF) of experimental XANES spectra was performed in the range of 12630 to 12850 eV using a library of 52 standard selenocompounds (Fakra et al., 2018). The best LCF was obtained by minimizing the normalized sum of squares residuals (NSS = 0 = perfect fit). The error margin for the fraction found for selenocompounds is ±10%. Additionally, valence-state scatter plots, where each datapoint represent a XANES spectrum, were obtained for quick visualization of Se valence state in the samples, following procedures detailed in Fakra et al. (2018).

## Results

### Interference of sulfur/phosphorus with short-term se uptake and translocation

**Figure 1:**
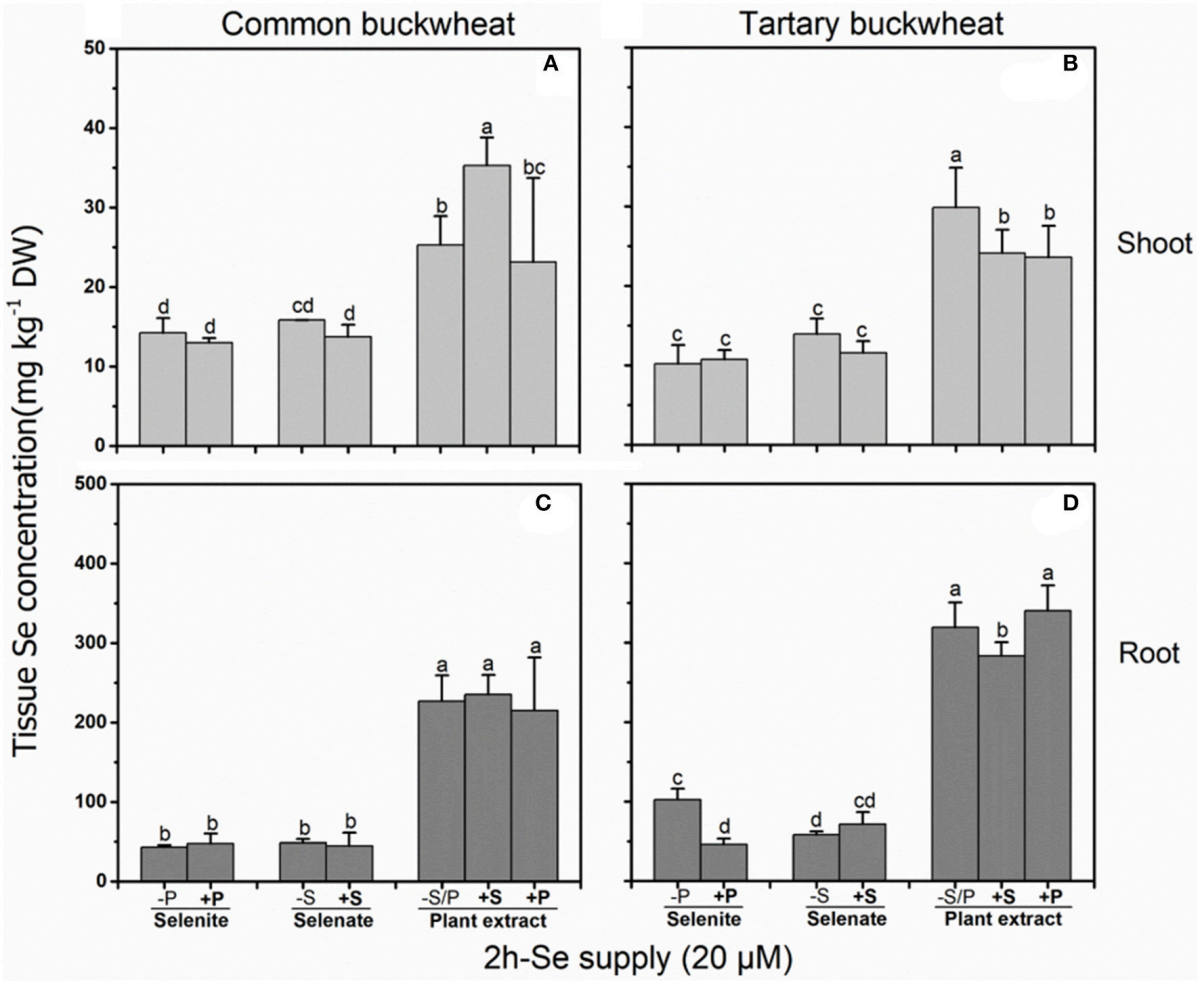
Shoot **(A,B)** and root **(C,D)** selenium (Se) accumulation in 2-week-old Common buckwheat **(A,C)** and Tartary buckwheat **(B,D)** plants incubated for 2 h with 20 μM of Se as selenite, selenate or *Astragalus bisulcatus* extract (methyl-SeCys) in the presence of either 0 or 0.5 mM of sulfate/phosphate. Values shown are the mean ± SE (*n* = 3). Different letters indicate significant difference between treatments (*P* < 0.05).

In a 2 h Se uptake study with buckwheat seedlings, the presence of 0.5 mM sulfate or phosphate both negatively affected (*P* < 0.05) Se accumulation from *A. bisulcatus* plant extract (i.e., methyl-SeCys) in the shoot of tartary buckwheat, while in common buckwheat sulfate enhanced shoot Se concentration from methyl-SeCys and phosphate had no effect (Figure 1B). In roots, Se accumulation from plant extract was reduced by sulfate, but only for tartary buckwheat (Figures 1C,D). Shoot and root Se accumulation from selenate were not affected by sulfate supply in either species, and phosphate only inhibited Se accumulation from selenite in the root of tartary buckwheat (Figure 1D).

In this 2 h uptake study, similar Se concentrations were found in shoots of common buckwheat and tartary buckwheat supplied with either selenate or selenite (10–15 mg kg^−1^ DW), whereas 2- to 3-fold higher Se levels were observed in shoots of both buckwheat species supplied with the same Se concentration as *A. bisulcatus* plant extract (i.e., methyl-SeCys). Likewise, Se levels in roots were 4- to 6-fold higher in common buckwheat and 2- to 7-fold higher in tartary buckwheat when supplied with plant extract (250–350 mg kg^−1^ DW) as compared to selenite and selenate (50–100 mg kg^−1^ DW). The levels of Se in the root of both species of buckwheat were higher than those in the shoot, for all three Se treatments and regardless of S or P supply (Figure 1).

**Figure 2:**
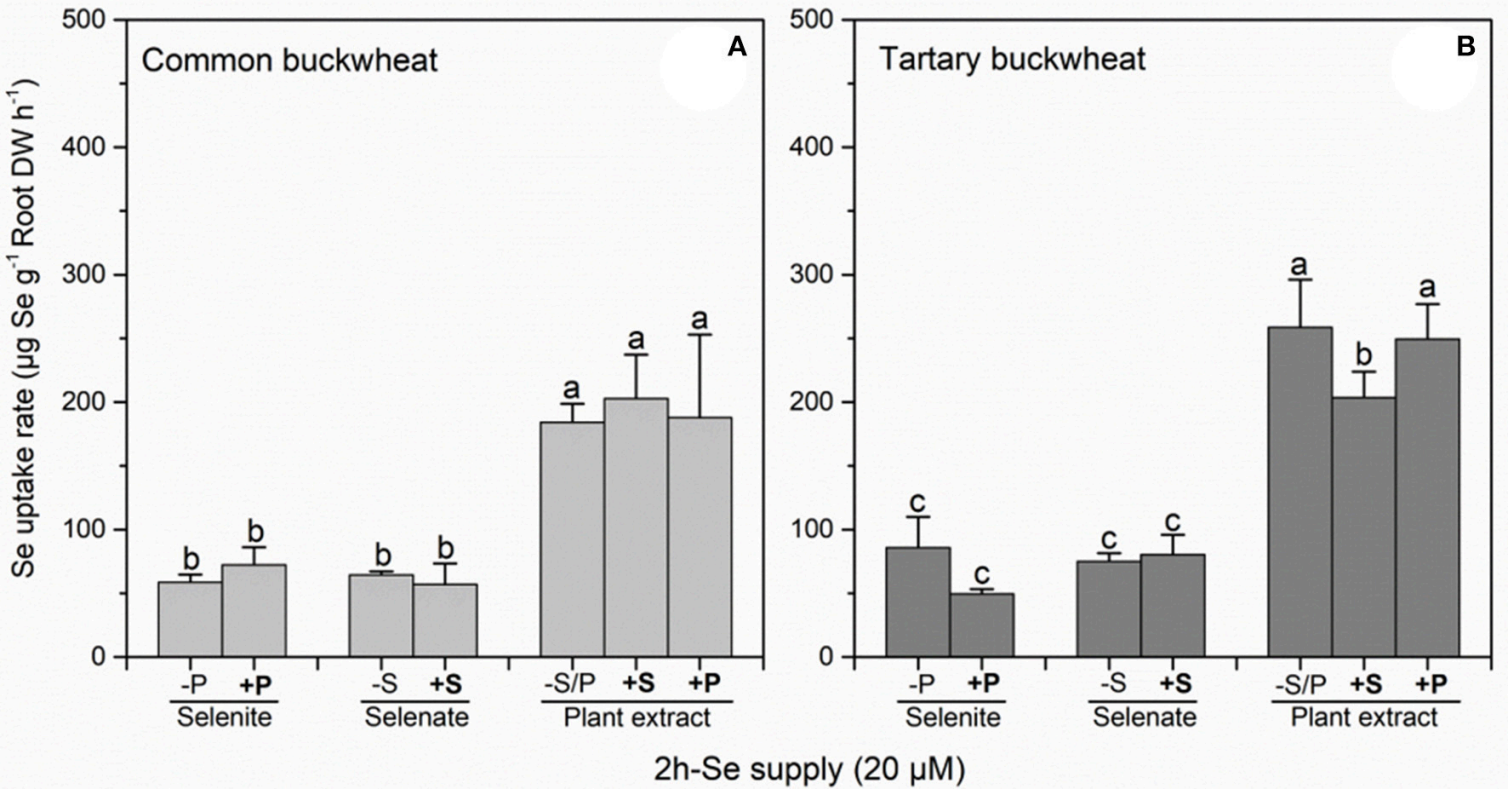
Root Se uptake rate of Common buckwheat **(A)** and Tartary buckwheat **(B)** plants incubated for 2 h with 20 μM of Se as selenite, selenite, and *Astragalus bisulcatus* plant extract (methyl-SeCys) in the presence of either 0 or 0.5 mM of sulfate or phosphate. Values shown are the mean ± SE (*n* = 3). Different letters indicate significant difference between treatments (*P* < 0.05).

Calculation of Se uptake rate for common buckwheat and tartary buckwheat in relation to the three Se forms and S/P supplement revealed a significant inhibitory effect of 0.5 mM sulfate supply on plant extract Se uptake rate in tartary buckwheat but not in common buckwheat (Figure 2). Approximately 2- to 2.5-fold (common buckwheat) and 2- to 6-fold (tartary buckwheat) higher Se uptake rates were observed from plant extract Se (methyl-SeCys), compared to selenite or selenate, respectively.

Calculation of Se translocation factor (TF) from root to shoot revealed a difference between the two buckwheat species (Table 1): common buckwheat showed higher Se translocation to the shoot under all treatments except for plant extract Se without S or P. Furthermore, for the selenite treatments, the presence of 0.5 mM phosphate enhanced TF values 1.2-fold for common buckwheat and 1.8-fold for tartary buckwheat. The sulfate supplement did not reveal any effects on the TF of selenate in common buckwheat, but a 0.2-fold reduction of Se TF was seen after the addition of sulfate in tartary buckwheat. Furthermore, the presence of S and P led to a slight increase in Se TF of common buckwheat for the plant extract Se application (1.1-fold and 1.2-fold, respectively), while TF values of tartary buckwheat under plant extract Se treatments were somewhat decreased by the addition of S and P, by 1.3-fold and 1.2-fold, respectively (Table 1).

**Table 1:** The Se translocation factor (TF) of Common buckwheat and Tartary buckwheat after 2 h of exposure to 20 μM of different forms of Se, with/without 0.5 mM sulfate or phosphate supplement.

	**Selenite**		**Selenate**		***A. bis*** **extract (Methyl-SeCys)**		
	**–P**	**+P**	**–S**	**+S**	**–S/P**	**+S**	**+P**
Common buckwheat	1.74 ± 0.36	2.13 ± 0.50	1.67 ± 0.15	1.69 ± 0.60	0.63 ± 0.12	0.72 ± 0.11	0.74 ± 0.09
Tartary buckwheat	0.66 ± 0.25	1.16 ± 0.19	1.58 ± 0.31	1.27 ± 0.21	0.62 ± 0.15	0.44 ± 0.08	0.47 ± 0.02

*Values shown are the mean ± SE (n = 3)*.

### Effect of se speciation on se uptake and assimilation over 24 h in mature plants

**Figure 3:**
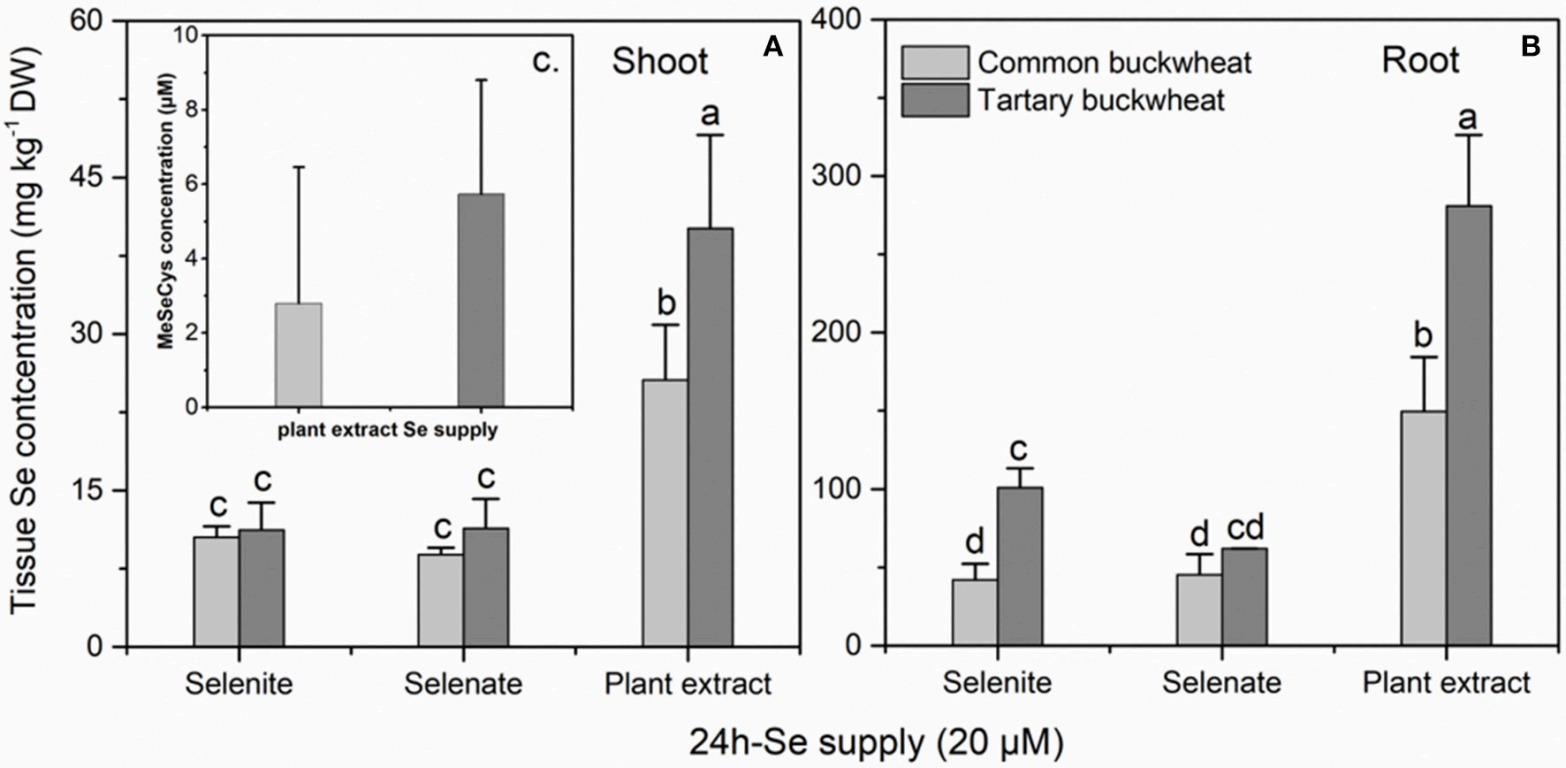
Shoot **(A)** and root **(B)** selenium (Se) accumulation of 30-day-old Common buckwheat and Tartary buckwheat plants incubated for 24 h with 20 μM of selenite, selenate or *Astragalus bisulcatus* plant extract (methyl-SeCys). **(C)** Methyl-SeCys concentration in xylem sap of common buckwheat and tartary buckwheat fed with plant extract Se. Values shown are the mean ± SE (*n* = 3). Different letters indicate significant difference between treatments (*P* < 0.05).

In a longer-term (24 h) uptake study performed at the plant flowering stage, the Se concentration in shoot and root were significantly higher for both buckwheat species when supplied with plant-extracted Se methyl-SeCys as compared to selenite or selenate (Figures 3A,B). Additionally, at this plant stage the tartary buckwheat plants accumulated more Se than common buckwheat for several treatments, tartary buckwheat Se levels were higher in root and shoot when supplied with plant extracted methyl-SeCys, and in roots when supplied with selenite. In agreement with this trend, after 24 h of exposure to 20 μM Se of the different Se forms, methyl-SeCys levels in xylem sap were on average 2-fold higher for tartary buckwheat than for common buckwheat (Figure 3C, *P* < 0.05). Methyl-SeCys was the only form of organic Se detected in the xylem sap samples from both species supplied with plant extract. No Se was detected in xylem sap from plants supplied with selenate or selenite, but it should be noted that inorganic Se is not measured in this LC-MS analysis.

### Effect of long-term se supply on dry matter production

**Figure 4:**
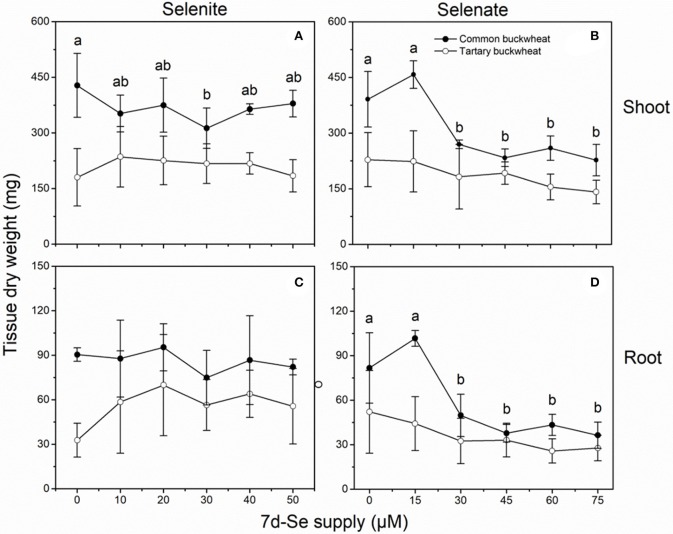
Shoot **(A,B)** and root **(C,D)** dry weight of 2-week-old Tartary buckwheat (closed circles) and Common buckwheat (open circles) plants incubated for 7 days with selenite or selenate. Different letters indicate significant differences among Se treatments within species (*P* < 0.05). Values shown are the mean ± SE (*n* = 3).

The shoot and root dry weight of common buckwheat were overall higher than those of the tartary buckwheat plantlets, after the 7 d uptake experiment (Figure 4). None of the selenite concentrations significantly inhibited growth of either species, but it is interesting to note that the average root and shoot DW of common buckwheat showed a slightly downward trend with increasing selenite concentration, while tartary buckwheat showed an increasing average DW production (Figures 4A,C). Compared to the control treatment without Se, approximately 1.7- to 2.2-fold higher root dry weight was found for tartary buckwheat after treatment with selenite, but there was no statistical difference (Figure 4C).

Selenate negatively affected biomass production for both species, and common buckwheat was clearly more affected than tartary buckwheat (Figures 4B,D). Exposure for 7d to levels above 20 μM selenate inhibited shoot and root dry matter production of common buckwheat by 30–40% and 40–55%, respectively, while average shoot and root DW of tartary buckwheat were only marginally lower. It is interesting to note that the lowest (15 μM) selenate treatment resulted in a small increase in shoot (17%) and root (25%) dry weight for common buckwheat (Figures 4B,D).

### Long-term se accumulation capacity in both buckwheat species

**Figure 5:**
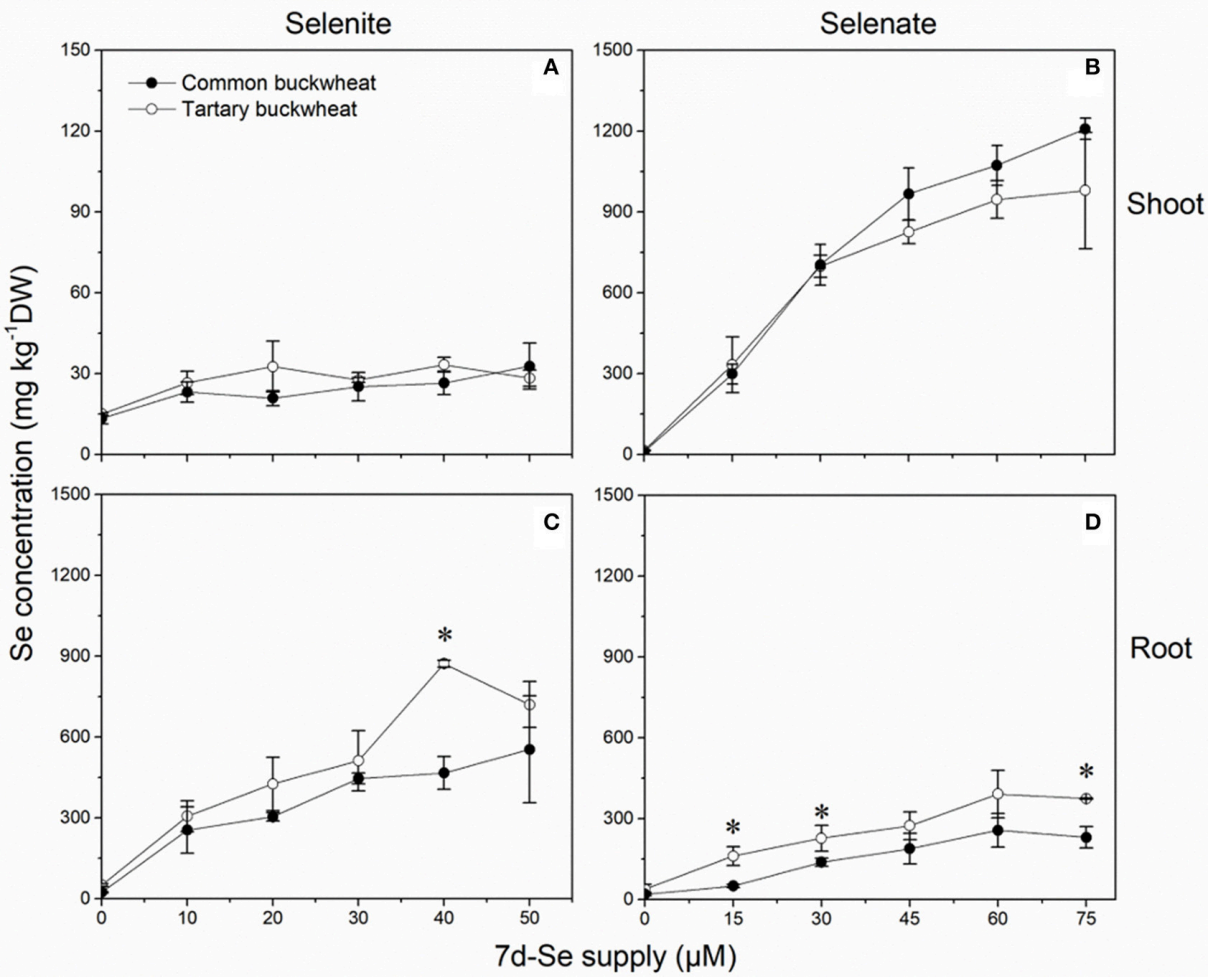
Shoot **(A,B)** and root **(C,D)** selenium (Se) accumulation of 2-week-old Common buckwheat (closed circles) and Tartary buckwheat (open circles) plants incubated for 7 days with selenite or selenate. The asterisks denote significant differences between the two species of buckwheat under the same Se treatment (*P* < 0.05). Values shown are the mean ± SE (*n* = 3).

Selenium accumulation capacity in both buckwheat species were detected by exposing 2-week old plantlets for a period of 7 days to a range of selenite and selenate concentrations (Figure 5). When exposed to selenite, both species accumulated 10- to 30-fold higher Se levels in root than shoot, and tartary buckwheat generally reached somewhat higher Se levels than common buckwheat, particularly in root (Figures 5A,C). After 7 days of selenate treatment, on the other hand, both species accumulated more Se in shoot than root, and differed from each other in that common buckwheat accumulated more Se in its shoot and less in its root than tartary buckwheat (Figures 5 B,D). This difference is also very apparent from the calculated TF, which was up to 2.5-fold higher for common buckwheat under selenate treatment (Table 2). Incidentally, for selenite treated plants, the TF was also higher for common buckwheat (Table 2), due to the higher root Se levels in tartary buckwheat and roughly equal shoot Se levels in both species. There was evidence of Se toxicity in both species after exposure to selenate above 30 μM or even above 15 μM. In contrast, there was no apparent toxicity in either species under selenite treatment for the same period.

**Table 2:** The Se translocation factor (TF) of Common buckwheat and Tartary buckwheat after 7-day of exposure to selenite and selenate.

**Form of Se supplied**	**Supplied Se conc. (μM)**	**Common buckwheat**	**Tartary buckwheat**
Selenite	10	0.39 ± 0.02	0.39 ± 0.10
	20	0.22 ± 0.06	0.26 ± 0.07
	30	0.24 ± 006	0.22 ± 0.07
	40	0.20 ± 0.05	0.13 ± 0.02
	50	0.28 ± 0.05	0.14 ± 0.04
Selenate	15	27.72 ± 7.51	11.33 ± 5.35
	30	28.89 ± 7.18	17.51 ± 2.84
	45	32.92 ± 3.88	18.35 ± 1.62
	60	26.30 ± 7.22	15.16 ± 3.19
	75	33.52 ± 4.46	13.54 ± 2.71

*Values shown are the mean ± SE (n = 3)*.

### Interaction of long-term se supply with S and P accumulation

Selenite treatment had overall a slight negative effect on S accumulation in both buckwheat species, which was more apparent in the shoot for common buckwheat and more in the root for tartary buckwheat (Table 3). The two buckwheat species had similar S levels under these conditions. Selenate showed a significant positive effect on shoot S accumulation in both species, the S concentration in shoot of common buckwheat and tartary buckwheat was 2.3- to 3.3- fold and 2.6- to 4.0-fold enhanced under selenate supply. In the roots of both buckwheat species, S accumulation was not much influenced by selenate treatment; overall higher S levels were found in tartary buckwheat roots compared to common buckwheat (Table 3).

**Table 3:** Shoot and root sulfur (S) accumulation of 2-week-old Tartary buckwheat and Common buckwheat plants incubated for 7 days with either selenite or selenate.

**Form of Se supplied**	**Supplied Se conc. (μM)**	**Common buckwheat**		**Tartary buckwheat**	
		**Shoot**	**Root**	**Shoot**	**Root**
Selenite	0	3.44 ± 0.34a	3.31 ± 0.23	2.90 ± 0.87	3.39 ± 0.79a
	10	2.54 ± 0.16b	3.31 ± 0.34	2.66 ± 0.44	3.14 ± 0.33a
	20	2.89 ± 0.86ab	3.12 ± 0.59	2.72 ± 0.44	2.38 ± 0.26b
	30	2.60 ± 0.43ab	2.51 ± 0.19	2.49 ± 0.72	2.35 ± 0.30b
	40	2.32 ± 0.14b	2.87 ± 1.23	2.33 ± 0.38	3.50 ± 0.29a
	50	2.78 ± 0.50ab	2.48 ± 0.16	2.38 ± 0.36	3.31 ± 0.10a
Selenate	0	3.17 ± 0.34c	2.56 ± 0.28a	2.65 ± 0.16c	3.69 ± 0.31cd
	15	7.49 ± 1.57b	1.75 ± 0.11b	10.5 ± 3.72a	4.29 ± 0.4ab
	30	10.59 ± 0.62a	2.59 ± 0.18a	10.28 ± 0.59a	4.38 ± 0.15a
	45	7.57 ± 1.42b	2.42 ± 0.25a	9.23 ± 0.32ab	4.11 ± 0.23abc
	60	7.42 ± 1.37b	2.75 ± 0.50a	7.96 ± 1.34ab	3.83 ± 0.33bcd
	75	7.17 ± 0.89b	2.54 ± 0.27a	7.01 ± 1.75b	3.34 ± 0.14d

*Values shown are the mean ± SE (n = 3). Different letters indicate statistically different means among Se level treatments within species (P < 0.05)*.

The level of P in the shoot of both buckwheat species was not found to significantly differ in response to increased selenite concentration in the nutrient solution, and tartary buckwheat showed higher P accumulation in shoot compared to common buckwheat (Table 4). Increasing selenate supply led to higher shoot P levels in both buckwheat species, and tartary buckwheat again had higher P levels than common buckwheat. Root P levels were overall lower than those in shoots, and tartary buckwheat again tended to have higher levels than common buckwheat (Table 4). Selenate treatment had a small but significant negative effect on root P levels for tartary buckwheat, but not for common buckwheat; selenite did not have a clear, consistent effect on root P levels for either species (Table 4).

**Table 4:** Shoot and root phosphorus (P) accumulation of 2-week-old Tartary buckwheat and Common buckwheat plants incubated for 7 days with selenite or selenate.

**Form of Se supplied**	**Supplied Se conc. (μM)**	**Common buckwheat**		**Tartary buckwheat**	
		**Shoot**	**Root**	**Shoot**	**Root**
Selenite	0	5.30 ± 0.66	3.28 ± 0.31bc	8.82 ± 0.78	3.5 ± 0.46
	10	5.75 ± 0.25	3.62 ± 0.57bc	7.91 ± 1.56	3.49 ± 0.26
	20	5.34 ± 1.12	3.42 ± 0.77c	7.6 ± 1.17	3.02 ± 0.47
	30	5.13 ± 0.83	2.95 ± 0.22c	7.35 ± 1.84	3.01 ± 0.68
	40	5.08 ± 0.15	3.37 ± 1.51a	7.5 ± 0.51	4.34 ± 0.16
	50	4.75 ± 1.21	2.5 ± 0.12ab	8.15 ± 1.57	3.95 ± 0.26
Selenate	0	5.78 ± 1.00cd	2.47 ± 0.20	8.54 ± 0.54b	4.14 ± 0.57a
	15	5.16 ± 0.45d	2.55 ± 0.29	8.32 ± 0.62b	4.06 ± 0.15a
	30	8.26 ± 0.98a	2.82 ± 0.55	9.38 ± 0.93ab	3.15 ± 0.47b
	45	7.14 ± 0.66ab	2.6 ± 0.20	9.89 ± 1.18ab	3.5 ± 0.19ab
	60	6.56 ± 0.94bc	2.5 ± 0.50	10.97 ± 2.10a	3.6 ± 0.36ab
	75	6.72 ± 0.11bc	2.47 ± 0.31	10.76 ± 0.48a	3.14 ± 0.32b

*Values shown are the mean ± SE (n = 3). Different letters indicate statistically different means among Se level treatments within species (P < 0.05)*.

### Following the fate of se after two-time supply with different selenocompounds

**Figure 6:**
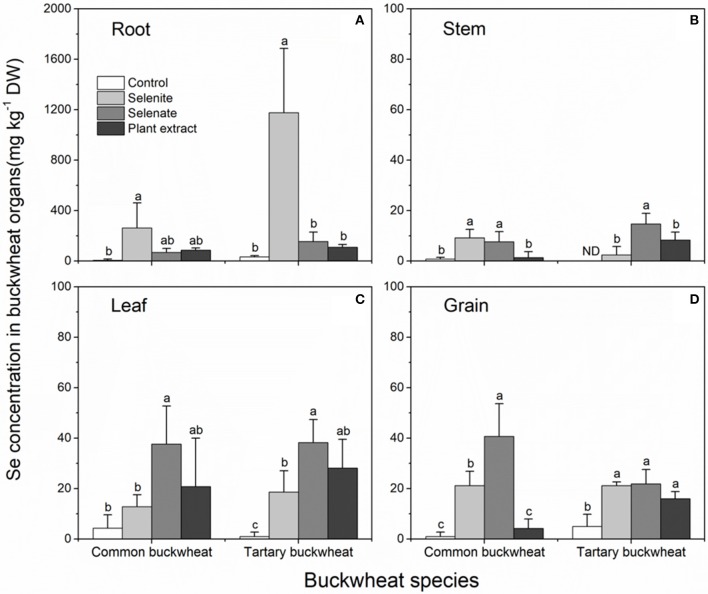
Selenium accumulation in the organs **(A)** root; **(B)** stem; **(C)** leaf; **(D)** grain; of Common buckwheat and Tartary buckwheat treated with 20 μM Se of selenite, selenate, or *Astragalus bisulcatus* extract (methyl-SeCys) twice during the growth period. Different letters above bars indicate statistically different means among Se species treatments within species (*P* < 0.05).

To investigate the fate of the different selenocompounds all the way to plant maturity, both species were grown until maturity (seed set) and supplied twice during their lifetime with 20 μM Se as selenite, selenate or as *A. bisulcatus* plant extracted methyl-SeCys, with a 15-day interval. This Se supply resulted in measurably elevated Se levels in all plant organs, but to different extent depending on organ, species, and form of Se supplied (Figure 6). In the roots of both species, the Se concentration was much higher for the selenite application compared to the other treatments, and tartary buckwheat accumulated overall higher root Se levels than common buckwheat, from all three forms of Se supplied (Figure 6A). The Se concentrations in the stem were the lowest among all organs, for all Se treatments (Figure 6B). Compared to tartary buckwheat, common buckwheat accumulated more Se in its stem when supplied with selenite, but less when supplied with plant extract (Figure 6B). The leaf Se levels were clearly elevated by the Se treatments, and comparable for both buckwheat species (Figure 6C). The levels ranged from 15 to 20 mg Se kg^−1^ DW for selenite-supplied plants to 40 mg kg^−1^ DW for selenate-supplied plants; plants supplied with *A. bisulcatus* plant extract showed intermediate leaf Se levels (Figure 6C).

The Se accumulation in the grain (seed) of buckwheat deserves special interest, as the edible part for humans. The seed Se levels were overall similar to those found in the leaves, and in all but one treatment well above the background Se levels found under control conditions (Figure 6D). Common buckwheat exhibited the highest seed Se accumulation in plants that received selenate (40 mg kg^−1^ DW), which were 2-fold higher than in plants that had received selenite, while levels in plants supplied with plant extract were not above background. In tartary buckwheat grain, all three forms of Se increased the Se content significantly and equally, to ~20 mg kg^−1^ DW (Figure 6D).

**Figure 7:**
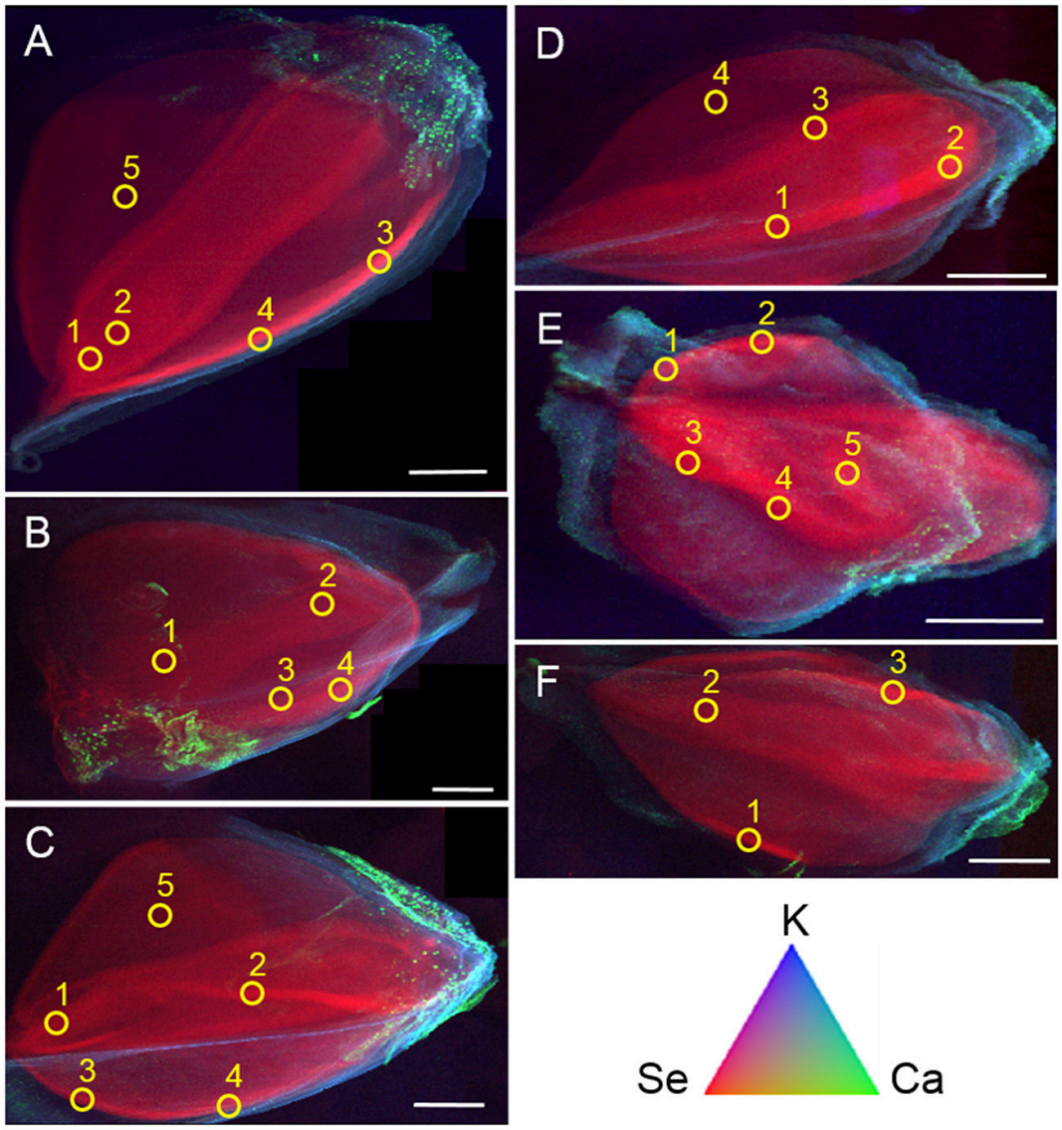
Tricolor coded micro-focused X-ray fluorescence (XRF) maps of seeds from common buckwheat **(A–C)** and tartary buckwheat **(D–F)** after twice biofortification with 20 μM Se as selenate **(A,D)**, selenite **(B,E)** or *Astragalus bisulcatus* extract **(C,F)**. Selenium is shown in red, calcium in green and potassium in blue. Yellow circles denote locations where XANES spectra were collected, to determine Se speciation. All scale bars are 1 mm.

**Figure 8:**
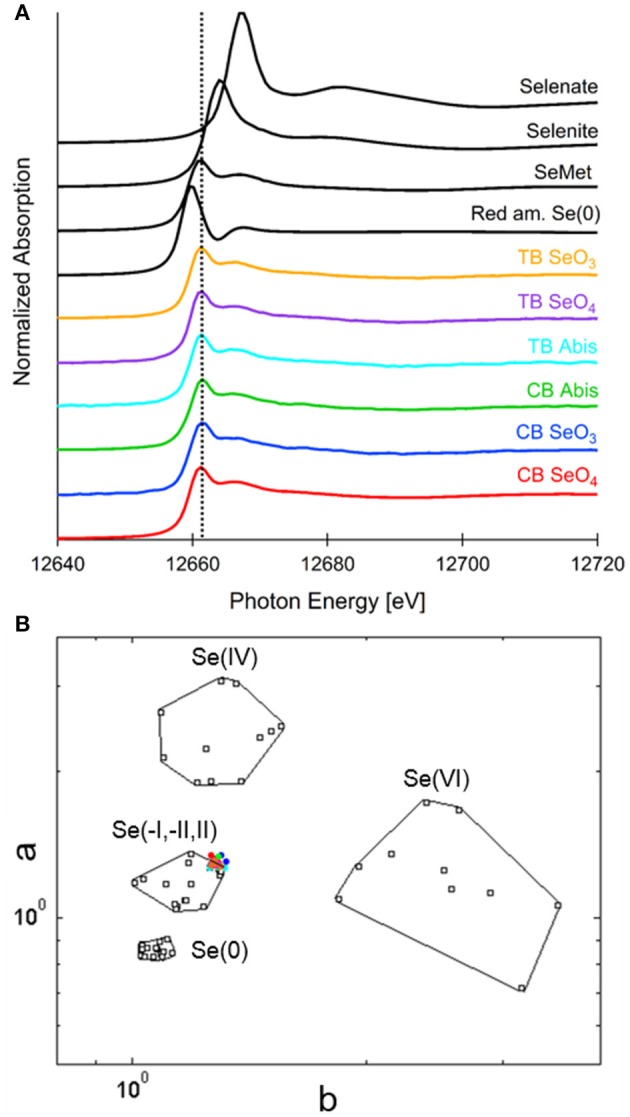
Selenium speciation in seeds of common buckwheat and tartary buckwheat supplied with selenate, selenite or *A. bisulcatus* extract. **(A)** Average XANES spectra for each sample, in comparison with Se standards: a C-Se-C compound, selenomethionine, as well as red amorphous Se(0), sodium selenate and sodium selenite. **(B)** Se valence-state scatter plot shows that all XANES spectra plot in close proximity to organic Se standards. Se standards are in black. Colored dots coding is as follows: red, CB SeO4; blue, CB SeO3; green, CB Abis extract; magenta, TB SeO4; orange, TB SeO3; cyan, TB Abis extract.

Seeds from both plant species treated with the three different selenocompounds were analyzed for their Se distribution and chemical speciation using X-ray microprobe. XRF mapping revealed that Se (Figure 7, shown in red) was concentrated in the embryo within each seed for both species and all three Se treatments. The seed coat had little or no Se, but contained Ca and K (Figure 7). XANES spectra obtained at different locations within the embryo (indicated with yellow circles in Figure 7) were fitted to standard Se compounds. All seeds contained predominantly (96–100%) organic Se with a C-Se-C configuration, that best fitted with the standards selenomethionine, methyl-SeCys, and γ-glutamyl-methyl-SeCys (Figure 8).

## Discussion

This study explored the capacity of common buckwheat and tartary buckwheat to accumulate and tolerate Se as either selenite, selenate or as *A. bisulcatus*-extracted methyl-SeCys. The species were exposed to Se under controlled conditions at different life stages (seedling, plantlets, or until maturity) and for different durations (2 and 24 h, 7 days, 10 weeks). Both species were able to accumulate Se to significant and similar levels, up to ~1,000 mg kg^−1^ DW in shoots of selenate-treated plants and ~600 mg kg^−1^ DW in roots of selenite-treated plants after 7 days. Both species were tolerant to selenite up to the highest level tested, i.e., 50 μM. Tartary buckwheat was also quite tolerant to selenate (up to 75 μM), while common buckwheat showed growth reduction above 25 μM. In a study where plants were spiked with 20 μM Se twice over the duration of their lifetime, both species accumulated 15–40 mg Se kg^−1^ DW in seeds, leaves and stems, from all three selenocompounds. Regardless of plant species and form of Se supplied, the form of Se in seeds was organic Se with a C-Se-C configuration. This could be selenomethionine, methyl-SeCys, g-glutamyl-methyl-SeCys, other C-Se-C compounds not in our standards, or a combination thereof.

These findings are very relevant for Se biofortification applications. Organic forms of Se with a C-Se-C configuration, like those found in these buckwheat seeds are considered desirable for biofortification. Apparently, it does not matter which form of Se is supplied to buckwheat, it always stores C-Se-C compounds in its seeds. The levels found in this study would be considered high to be used as Se biofortified material. The daily recommended Se intake for adult humans is around 55–75 μg. If seeds contain 15 mg Se kg^−1^ DW, 4–5 g (a tablespoon) would provide sufficient Se to satisfy the daily requirement. Thus, lower Se supply would be preferable in a field setting than those used here. Another way to achieve optimal Se concentration in the final product would be to mix high-Se with low-Se buckwheat flour. Additionally, the high Se levels in seeds together with little growth reduction illustrates the higher tolerance of buckwheat to Se (Supplementary Figure 1), when compared with Se-sensitive tobacco and soybeans (Martin and Trelease, 1938) or even wheat (Lyons et al., 2005b).

There were some interesting differences with respect to how the plants processed the different forms of Se. Over a 24 h period, the Se in the *A. bisulcatus*-extract (methyl-SeCys) was taken up faster compared to selenite and selenate, and translocated via the xylem to the shoot. This indicates that the Se absorption mechanism of the methyl-SeCys in the plant extract was more efficient than those for the inorganic forms of Se. Indeed, organic forms of Se have been reported to show fast plant uptake rates through amino acid transporters (Kikkert and Berkelaar, 2013), compared to inorganic selenite and selenate uptake via phosphate and sulfate transporters, respectively (Li et al., 2008; El Mehdawi et al., 2018). In the long-term buckwheat biofortification study, the Se levels found in the plant tissues after methyl-SeCys supply were not higher and sometimes even lower than those for the inorganic forms of Se. This may point to a higher loss of Se via volatilization, when supplied with methyl-SeCys. This would be in agreement with earlier studies (Zayed and Terry, 1994; de Souza et al., 2000), which showed organic Se is volatilized much more readily than inorganic Se. From a practical perspective, biofortification with green manure containing methyl-SeCys is best timed shortly before harvest, to avoid loss via volatilization. Selenate and selenite also showed differences in accumulation pattern, with selenate being readily translocated to the shoot while selenite stayed in the root. The same pattern has been found for other species (Zayed and Terry, 1994). This low translocation to shoots is attributed to the rapid conversion of selenite to organic Se species in the roots (Kahakachchi et al., 2004; Inostroza-Blancheteau et al., 2013). With respect to the translocation of different forms of Se in buckwheat plants, common buckwheat showed a better capacity of Se translocation for all three Se speciation treatments, compared to the Se translocation ability of tartary buckwheat. Although the plants used in this study were in each experiment the same age for both species, common buckwheat commonly had attained more biomass than tartary buckwheat before the assay, which might explain its higher Se translocation factor, the bigger shoot of common buckwheat plants may have drawn up more Se due to transpiration (Kollman et al., 1974).

The interaction with sulfate or phosphate also differed for the selenocompounds. In the 2 h uptake study, accumulation of Se from *A. bisulcatus* plant extract was influenced by sulfate and phosphate, which both reduced accumulation in shoot for tartary buckwheat, while sulfate promoted Se accumulation in common buckwheat. It is not clear what the mechanism could be for these effects. Furthermore, uptake of selenite was inhibited by phosphate in the root of tartary buckwheat, which may be caused by competitive inhibition. Phosphate appeared to promote translocation of selenite-derived Se in both species, for reasons not readily apparent. It is surprising that selenate uptake was not inhibited by sulfate in the 2 h uptake study, in view of earlier results from other plant species (Schiavon et al., 2015; El Mehdawi et al., 2018) and evidence that selenate is taken up by sulfate transporters (El Kassis et al., 2007).

Tartary buckwheat showed improved growth over 7 days in the presence of selenite up to 40 μM, as did common buckwheat for selenate at 15 μM. These data are in agreement with earlier reports that low concentrations of selenate can benefit plant growth (Hartikainen, 2005; Pilon-Smits et al., 2009), due to activation of antioxidant mechanisms. Furthermore, there was a clear impact of selenate application on S and P distribution in plants (Supplementary Figures 2, 3), both species of buckwheat accumulated more S overall, and translocated more S and P from root to shoot, which was also observed in wheat cultivated in high Se levels of soil or solution conditions (Lyons et al., 2005b), it might help resist Se stress (Feng et al., 2013). This finding suggests that low level Se supplementation could enhance crop nutritional quality not only because of the Se itself, but also by enhancing the levels of these other nutrients (Malagoli et al., 2015).

Buckwheat is a crop that grows well in low-Se areas of China and Europe. These buckwheat Se experiments show that Se application via the roots in different forms is a very efficient way to enhance the Se concentration in all plant organs, including the seeds. The form of Se in seeds was organic with a C-Se-C configuration in all cases, most like the selenomethionine standard, which is similar to cereals reported in the previous finding showing Se concentration to be highest in the embryo of wheat (mostly as selenomethionine) (Lyons et al., 2005a). Selenomethionine and other C-Se-C compounds are considered most desirable as a source of Se for mammals, making buckwheat an attractive species for Se biofortification.

## Author contributions

YJ, EP-S, HQ, and YH designed and performed most of the experiments, analyzed the data and wrote the manuscript. AE, Tripti, LL, and GS helped with the data collection and analysis, editing of the manuscript as well. SF helped to perform the X-ray microprobe imaging. All authors read and approved the manuscript.

### Conflict of interest statement

The authors declare that the research was conducted in the absence of any commercial or financial relationships that could be construed as a potential conflict of interest.

## References

[B1] Abrams M. M. Shennan C. Zasoski R. J. Burau R. G. (1990). Selenomethionine uptake by wheat seedlings. Agron. J. 82, 1127–1130. 10.2134/agronj1990.00021962008200060021x

[B2] Bañuelos G. S. Arroyo I. Pickering I. J. Yang S. I. Freeman J. L. (2015). Selenium biofortification of broccoli and carrots grown in soil amended with Se-enriched hyperaccumulator *Stanleya pinnata*. Food Chem. 166, 603–608. 10.1016/j.foodchem.2014.06.071 25053099

[B3] Bañuelos G. S. Arroyo I. S. Dangi S. R. Zambrano M. C. (2016). Continued selenium biofortification of carrots and broccoli grown in soils once amended with Se-enriched S. pinnata. Front. Plant Sci. 7:1251. 10.3389/fpls.2016.01251 27602038 PMC4993952

[B4] Bendich A. (2001). Dietary reference intakes for vitamin C, vitamin E, selenium, and carotenoids institute of medicine washington, DC: National Academy Press, 2000 ISBN: 0-309-06935-1. Nutrition 17:364 10.1016/S0899-9007(00)00596-7

[B5] Broadley M. R. White P. J. Bryson R. J. Meacham M. C. Bowen H. C. Johnson S. E.. (2006). Biofortification of UK food crops with selenium. Proc. Nutr. Soc. 65, 169–181. 10.1079/PNS2006490 16672078

[B6] Combs G. F. Jr. (2001). Selenium in global food systems. Brit. J. Nutr. 85, 517–547. 10.1079/BJN2000280 11348568

[B7] de Souza M. P. Pilon-Smits E. A. H. Terry N. (2000). The physiology and biochemistry of selenium volatilization by plants in Phytoremediation of Toxic Metals: Using Plants to Clean up the Environment, eds Ensley B. D. Raskin I. (New York , NY: Wiley and Sons), 171–190.

[B8] Dumont E. Ogra Y. Vanhaecke F. Suzuki K. T. Cornelis R. (2006). Liquid chromatography-mass spectrometry (LC-MS): a powerful combination for selenium speciation in garlic (*Allium sativum*). Anal. Bioanal. Chem. 384, 1196–1206. 10.1007/s00216-005-0272-6 16435092

[B9] El Kassis E. Cathala N. Rouached H. Fourcroy P. Berthomieu P. Terry N.. (2007). Characterization of a selenate-resistant *Arabidopsis* mutant. Root growth as a potential target for selenate toxicity. Plant Physiol. 143, 1231–1241. 10.1104/pp.106.091462 17208959 PMC1820920

[B10] El Mehdawi A. F. Cappa J. J. Fakra S. C. Self J. Pilon-Smits E. A. H. (2012). Interactions of selenium hyperaccumulators and nonaccumulators during cocultivation on seleniferous or nonseleniferous soil - the importance of having good neighbors. New Phytol. 194, 264–277. 10.1111/j.1469-8137.2011.04043.x 22269105

[B11] El Mehdawi A. F. Jiang Y. Guignardi Z. S. Esmat A. Pilon M. Pilon-Smits E. A. H.. (2018). Influence of sulfate supply on selenium uptake dynamics and expression of sulfate/selenate transporters in selenium hyperaccumulator and nonhyperaccumulator Brassicaceae. New Phytol. 217, 194–205. 10.1111/nph.14838 29034966

[B12] Fakra S. C. Luef B. Castelle C. J. Mullin S. W. Williams K. H. Marcus M. A.. (2018). Correlative cryogenic spectromicroscopy to investigate selenium bioreduction products. Environ. Sci. Technol. 52, 503–512. 10.1021/acs.est.5b01409 26371540

[B13] Fassel V. A. (1978). Quantitative elemental analyses by plasma emission spectroscopy. Science 202, 183–191. 10.1126/science.202.4364.183 17801907

[B14] Feng R. Wei C. Tu S. (2013).The roles of selenium in protecting plants against abiotic stresses. Environ. Exp. Bot. 87, 58–68. 10.1016/j.envexpbot.2012.09.002

[B15] Golob A. GadŽo D. Stibilj V. Djikić M. Gavrić T. Kreft I.. (2016). Sulphur interferes with selenium accumulation in Tartary buckwheat plants. Plant Physiol. Biochem. 108, 32–36. 10.1016/j.plaphy.2016.07.001 27404132

[B16] Graham R. D. Welch R. M. Saunders D. A. Ortiz-Monasterio I. Bouis H. E. Bonierbale M. (2007). Nutritious subsistence food systems. Adv. Agron. 92, 1–74. 10.1016/S0065-2113(04)92001-9

[B17] Hartikainen H. (2005). Biogeochemistry of selenium and its impact on food chain quality and human health. J. Trace Elem. Med. Biol. 18, 309–318. 10.1016/j.jtemb.2005.02.009 16028492

[B18] Hasanuzzaman M. Hossain M. A. Fujita M. (2010). Selenium in higher plants: physiological role, antioxidant metabolism and abiotic stress tolerance. J. Plant Sci. 5, 354–375. 10.3923/jps.2010.354.375

[B19] Hoagland D. Arnon D. I. (1950). The water culture method for growing plants without soil. Calif . Agric. Exp. Stat. Circ. 347,1–32.

[B20] Inostroza-Blancheteau C. Reyes-Díaz M. Alberdi M. Godoy K. Rojas-Lillo Y. Cartes P. (2013). Influence of selenite on selenium uptake, differential antioxidant performance and gene expression of sulfate transporters in wheat genotypes. Plant Soil 369, 47–59. 10.1007/s11104-012-1492-0

[B21] Jiang Y. Zeng Z. H. Bu Y. Ren C. Z. Li J. Z. Han J. J. (2015). Effects of selenium fertilizer on grain yield, se uptake and distribution in common buckwheat (*Fagopyrum esculentum* Moench). Plant Soil Environ. 61, 371–377. 10.17221/284/2015-PSE

[B22] Jones G. D. Droz B. Greve P. Gottschalk P. Poffet D. McGrath S. P.. (2017). Selenium deficiency risk predicted to increase under future climate change. Proc. Natl. Acad. Sci. U.S.A. 114, 2848–2853. 10.1073/pnas.1611576114 28223487 PMC5358348

[B23] Kahakachchi C. Boakye H. T. Uden P. C. Tyson J. F. (2004). Chromatographic speciation of anionic and neutral selenium compounds in Se-accumulating *Brassica juncea* (Indian mustard) and in selenized yeast. J. Chromatogr. A. 1054, 303–312. 10.1016/S0021-9673(04)01287-7 15553157

[B24] Kikkert J. Berkelaar E. (2013). Plant uptake and translocation of inorganic and organic forms of selenium. Arch. Environ. Contam. Toxicol. 65, 458–465. 10.1007/s00244-013-9926-0 23793939

[B25] Kollman G. E. Streeter J. G. Jeffers D. L. Curry R. B. (1974). Accumulation and distribution of mineral nutrients, carbohydrate, and dry matter in soybean plants as influenced by reproductive sink size. Agron. J. 66, 549–554. 10.2134/agronj1974.00021962006600040021x

[B26] Li H. F. McGrath S. P. Zhao F. J. (2008). Selenium uptake, translocation and speciation in wheat supplied with selenate or selenite. New Phytol. 178, 92–102. 10.1111/j.1469-8137.2007.02343.x 18179602

[B27] Lyons G. H. Genc Y. Stangoulis J. C. Palmer L. T. Graham R. D. (2005a). Selenium distribution in wheat grain, and the effect of postharvest processing on wheat selenium content. Biol. Trace Elem. Res. 103, 155–168. 10.1385/BTER:103:2:155 15772439

[B28] Lyons G. H. Stangoulis J. C. R. Graham R. D. (2005b). Tolerance of wheat (*Triticum aestivum*, L.) to high soil and solution selenium levels. Plant Soil 270, 179–188. 10.1007/s11104-004-1390-1

[B29] Malagoli M. Schiavon M. Dall'Acqua S. Pilon-Smits E. A. (2015). Effects of selenium biofortification on crop nutritional quality. Front. Plant Sci. 6:280. 10.3389/fpls.2015.00280 25954299 PMC4404738

[B30] Marcus M. A. Macdowell A. A. Celestre R. Manceau A. Miller T. Padmore H. A.. (2004). Beamline 10.3.2 at als: a hard x-ray microprobe for environmental and materials sciences. J. Synchrot. Radiat. 11, 239–247. 10.1107/S0909049504005837 15103110

[B31] Martin A. L. Trelease S. F. (1938). Absorption of selenium by tobacco and soy beans in sand cultures. Am. J. Bot. 25, 380–385. 10.2307/2436764

[B32] Navarro-Alarcon M. Cabrera-Vique C. (2008). Selenium in food and the human body: a review. Sci. Total Environ. 400, 115–141. 10.1016/j.scitotenv.2008.06.024 18657851

[B33] Pilon-Smits E. A. H. Quinn C. F. Tapken W. Malagoli M. Schiavon M. (2009). Physiological functions of beneficial elements. Curr. Opin. Plant Biol. 12, 267–274. 10.1016/j.pbi.2009.04.009 19477676

[B34] Rayman M. (2012). Selenium and human health. Lancet 379, 1256–1268. 10.1016/S0140-6736(11)61452-9 22381456

[B35] Schiavon M. Lima L. W. Jiang Y. Hawkesford M. J. (2017). Effects of selenium on plant metabolism and implications for crops and consumers in Selenium in Plants, eds Pilon-Smits E. A. H. Winkel L. H. E. Lin Z. Q. (Cham: Springer International Publishing), 257–275.

[B36] Schiavon M. Pilon M. Malagoli M. Pilon-Smits E. A. H. (2015). Exploring the importance of sulfate transporters and ATP sulfurylases for selenium hyperaccumulation-a comparison of *Stanleya pinnata* and *Brassica juncea* (Brassicaceae). Front. Plant Sci. 6:2 10.3389/fpls.2015.00002 25688247 PMC4304243

[B37] Schiavon M. Pilon-Smits E. A. H. (2017). The fascinating facets of plant selenium accumulation-biochemistry, physiology, evolution and ecology. New Phytol. 213, 1582–1596. 10.1111/nph.14378 27991670

[B38] Sors T. G. Ellis D. R. Salt D. E. (2005). Selenium uptake, translocation, assimilation and metabolic fate in plants. Photosynth. Res. 86, 373–389. 10.1007/s11120-005-5222-9 16307305

[B39] Stadtman T. C. (1974). Selenium biochemistry. Science 183, 915–922. 10.1126/science.183.4128.915 4605100

[B40] Terry N. Zayed A. M. de Souza M. P. Tarun A. S. (2000). Selenium in higher plants. Annu. Rev. Plant Physiol. Plant Molec. Biol. 51, 401–432. 10.1146/annurev.arplant.51.1.401 15012198

[B41] Valdez Barillas J. R. Quinn C. F. Freeman J. L. Lindblom S. D. Fakra S. C. Marcus M. A.. (2012). Selenium distribution and speciation in the hyperaccumulator *Astragalus bisulcatus* and associated ecological partners. Plant Physiol. 159, 1834–1844. 10.1104/pp.112.199307 22645068 PMC3425216

[B42] White P. J. Bowen H. C. Marshall B. Broadley M. R. (2007). Extraordinarily high leaf selenium to sulfur ratios define ‘Se-accumulator' plants. Ann. Bot. 100, 111–118. 10.1093/aob/mcm084 17525099 PMC2735298

[B43] Yasin M. El Mehdawi A. F. Jahn C. E. Anwar A. Turner M. F. S. Faisal M. (2014). Seleniferous soils as a source for production of selenium-enriched foods and potential of bacteria to enhance plant selenium uptake. Plant Soil 386, 385–394. 10.1007/s11104-014-2270-y

[B44] Zarcinas B. A. Cartwright B. Spouncer L. R. (1987). Nitric acid digestion and multi element analysis of plant material by inductively coupled plasmaspectrometry. Commun. Soil Sci. Plant Anal. 18, 131–146. 10.1080/00103628709367806

[B45] Zayed A. M. Terry N. (1994). Selenium volatilization in roots and shoots: effects of shoot removal and sulfate level. J. Plant Physiol. 143, 8–14. 10.1016/S0176-1617(11)82090-0

[B46] Zhang L. Hu B. Li W. Che R. Deng K. Li H.. (2014). OsPT2, a phosphate transporter, is involved in the active uptake of selenite in rice. New Phytol. 201, 1183–1191. 10.1111/nph.12596 24491113 PMC4284032

[B47] Zhu Y. G. Pilon-Smits E. A. Zhao F. J. Williams P. N. Meharg A. A. (2009). Selenium in higher plants: understanding mechanisms for biofortification and phytoremediation. Trends Plant Sci. 14, 436–442. 10.1016/j.tplants.2009.06.006 19665422

