# Supplementary material for: Characterization of Selenium Accumulation, Localization and Speciation in Buckwheat–Implications for Biofortification
Source: Front Plant Sci. 2018 Oct 31;9:1583. doi: 10.3389/fpls.2018.01583 (PMC6220067; doi:10.3389/fpls.2018.01583)
Supplement: Supplementary file 1 [file Table_1.docx]

Supplementary Material

**Characterization of Selenium Accumulation, Localization and Speciation in Buckwheat– Implications for Biofortification**

Ying Jiang^1,2^^,3^, Ali F. El Mehdawi^2^, Tripti^2,4^, Leonardo W. Lima^2^, Gavin Stonehouse^2^, Sirine C. Fakra^5^, Yuegao Hu^3^, Hua Qi^1^*, Elizabeth A. H. Pilon-Smits^2^*

^1^College of Agronomy, Shenyang Agricultural University, Shenyang, Liaoning 110866, China

^2^Department of Biology, Colorado State University, Fort Collins, CO 80523, USA

^3^College of Agronomy and biotechnology, China Agricultural University, Beijing 100193, China

^4^Department of Experimental Biology and Biotechnology, Institute of Natural Sciences, Ural Federal University, Ekaterinburg 620002, Russia

^5^Advanced Light Source, Lawrence Berkeley National Laboratory, Berkeley, CA 94720, USA

*** Correspondence:** Elizabeth Pilon-Smits: epsmits@colostate.edu；qihua10@163.com

# Supplementary Data (N.A.)

# Supplementary Figures and Tables

## Supplementary Figures



**Supplementary Figure 1.** Tissue Selenium/Sulfur ratio in shoot (a. and b.) and root (c. and d.) of 2-week-old Common buckwheat and Tartary buckwheat plants incubated for 7 days with selenite or selenate in Turface® with ¼ strength Hoagland solution. Different letters above bars indicate statistically different means among Se level treatments within species (*P* < 0.05). Values shown are the mean ± SE (n = 3).



**Supplementary Figure 2.** Tolerance index (ratio of total DW with Se to total DW without Se) of 2-week-old Common buckwheat and Tartary buckwheat plants incubated for 7 days with selenite or selenate. Different letters above bars indicate statistically different means among Se level treatments within species (*P* < 0.05). Values shown are the mean ± SE (n = 3).





**Supplementary Figure 3.** Tissue Se/P ratio in shoot (a. and b.) and root (c. and d.) of 2-week-old Common buckwheat and Tartary buckwheat plants incubated for 7 days with selenite or selenate in Turface ® with ¼ strength Hoagland solution. Different letters above bars indicate statistically different means among Se level treatments within species (*P* < 0.05). Values shown are the mean ± SE (n = 3).
